# Production and Characterization of a Novel Gluten-Free Fermented Beverage Based on Sprouted Oat Flour

**DOI:** 10.3390/foods10010139

**Published:** 2021-01-11

**Authors:** Natalia Aparicio-García, Cristina Martínez-Villaluenga, Juana Frias, Elena Peñas

**Affiliations:** Department of Food Characterization, Quality and Safety, Institute of Food Science, Technology and Nutrition (ICTAN-CSIC), 28006 Madrid, Spain; n.aparicio@ictan.csic.es (N.A.-G.); c.m.villaluenga@csic.es (C.M.-V.); frias@ictan.csic.es (J.F.)

**Keywords:** oat, sprouting, lactic acid fermentation, beverage, gluten-free

## Abstract

This study investigates the use of sprouted oat flour as a substrate to develop a novel gluten-free beverage by fermentation with a probiotic (*Lactobacillus plantarum* WCFS1) starter culture. Physicochemical, microbiological, nutritional and sensory properties of sprouted oat fermented beverage (SOFB) were characterized. After fermentation for 4 h, SOFB exhibited an acidity of 0.42 g lactic acid/100 mL, contents of lactic and acetic acids of 1.6 and 0.09 g/L, respectively, and high viable counts of probiotic starter culture (8.9 Log CFU/mL). Furthermore, SOFB was a good source of protein (1.7 g/100 mL), β-glucan (79 mg/100 mL), thiamine (676 μg/100 mL), riboflavin (28.1 μg/100 mL) and phenolic compounds (61.4 mg GAE/100 mL), and had a high antioxidant potential (164.3 mg TE/100 mL). Spoilage and pathogenic microorganisms were not detected in SOFB. The sensory attributes evaluated received scores higher than 6 in a 9-point hedonic scale, indicating that SOFB was well accepted by panelists. Storage of SOFB at 4 °C for 20 days maintained *L. plantarum* viability and a good microbial quality and did not substantially affect β-glucan content. SOFB fulfils current consumer demands regarding natural and wholesome plant-based foods.

## 1. Introduction

Whole grains are dietary staple foods consumed around the world due to their high nutritional quality. Among whole grains, oat has unique properties not found in other cereals, including its high content of soluble fiber (mainly β-glucan), essential fatty acids, vitamins and antioxidant phenolic compounds [1]. Due to its well-balanced nutritional profile and the wide range of phytochemicals contained in oat (*Avena sativa* L.), this grain is receiving particular popularity in recent years. An abundance of scientific evidence demonstrated that consumption of oat-based foods is associated with reduced risk of cardiovascular diseases, type 2 diabetes and obesity [2,3]. Oat does not naturally contain gluten, but it is frequently contaminated with gluten-containing cereals during cultivation, transportation and processing [4]. Due to its nutritional composition and health-promoting properties and its natural absence of gluten, oat represents an interesting raw material for developing innovative functional and gluten-free cereal-based foods.

The increasing consumer awareness for the relationship between nutrition and health, environment protection and animal rights together with pathological aspects such as lactose intolerance and cow’s milk allergy [5] have conducted to growing demand towards natural and healthy plant-based foods. Nowadays, plant-based fermented beverages are one of the most important segments within the functional food sector [6]. It has been largely demonstrated that lactic acid fermentation improves sensory characteristics, nutritional quality, texture and safety of starch-containing vegetables including cereals [7]. Strong scientific evidence support the beneficial effects of lactic acid bacteria fermentation in cereal matrices by enhancing the content and bioavailability of phenolic compounds, γ-aminobutyric acid (GABA) and antioxidant, anti-hypertensive and anti-inflammatory activities [8,9,10]. A number of recent investigations have evidenced that cereals are good fermentable substrates for lactic acid bacteria (LAB) that can be used as raw materials for development of probiotic beverages with enhanced nutritional properties [11,12,13,14,15]. Most of research studies focused on oat beverages have evaluated the influence of oat substrate amount, beverage formulation and type of starter culture on fermentation parameters, probiotic bacteria survival, technological and sensory properties and flavor and nutritional compounds of the resultant beverages [11,13,14,16,17]. However, to date, the development of functional fermented beverages from germinated oat has not been explored. Germination is a natural and economical process that improves the nutritional quality and content of bioactive compounds of grains [18,19]. Recent investigations performed by our group revealed that germination conditions have a huge influence on nutritional and functional features of germinated oat flour [20]. Germination parameters (temperature and time) were optimized to produce sprouted oat flours with enhanced content of protein, essential amino acids (Met, Cys and Phe), riboflavin, minerals, polyunsaturated fatty acids, as well as phenolic compounds, GABA and antioxidant activity [21]. These previous findings suggest the potential of sprouted oat flour to be used as ingredient for designing novel gluten-free functional foods. Hence, this study aimed to develop a gluten-free functional beverage from sprouted oat by lactic acid fermentation and to evaluate the physicochemical, nutritional, bioactive and sensory characteristics of the formulation obtained.

## 2. Materials and Methods

### 2.1. Chemical, Reagents and Standards

All chemicals, reagents and standards used were provided by Sigma-Aldrich (Madrid, Spain) unless otherwise specified. Gluten-free sucralose (Nutrisun GmbH & Co. KG, Seevetal, Germany) and sodium bicarbonate (Nortem Chem S. L., Cádiz, Spain) were purchased from a local supermarket.

### 2.2. Oat Substrate

Dehulled and gluten-free oat grains (variety Meeri) were provided by IBS Food Solutions (Barcelona, Spain). Seeds were harvested in Vaasa (Finland) at full ripening stage during the 2018–2019 crop year. Grains were stored under vacuum, in the dark at room temperature until their use. Oat grains were germinated at 18 °C for 4 days, as previously described [20]. These conditions have been selected since previous investigations performed by our group demonstrated that they maximized the nutritional and bioactive properties in the oat variety used in this study [20,21]. Sprouted oat grains were freeze-dried, milled using a coffee mill (Taurus, Oliana, Spain), passed through a 60-mesh sieve and stored in plastic bags under vacuum conditions at −20 °C.

The absence of gluten in oat grains and SOFB was confirmed by two commercial ELISA kits: Glutentox ELISA Competitivo (Biomedal, Seville, Spain) and INgezim Gluten Quick (Ingenasa, Madrid Spain).

### 2.3. Starter Culture

*Lactobacillus plantarum* WCFS1 was used as starter culture in this study based on their versatile metabolism and capacity of growing on different plant materials and also due to their probiotic characteristics [22]. The strain was kindly provided by Dr. Michiel Kleerebezem (NIZO Food Research, The Netherlands) and was kept in 50% glycerol (*v*/*v*) at −80 °C. The starter culture was obtained by overnight incubation of bacterial cells in Man Rogosa Sharpe (MRS; Pronadisa, Madrid, Spain) broth at 37 °C. Overnight bacterial cells were subcultured (1% *v*/*v*) in MRS for 4 h and subsequently they were harvested by centrifugation (8000 rpm, 10 min, 4 °C), washed twice and re-suspended in sterile distilled water to its original volume before inoculation in oat beverage.

### 2.4. Preparation of Sprouted Oat Fermented Beverage (SOFB)

Sprouted oat flour packed in plastic bags under vacuum conditions was heated at 90 °C for 30 min in a thermostatically controlled water bath to reduce the microbial load and eliminate the potential presence of the pathogen Bacillus cereus. A preliminary study aimed at optimizing the composition of SOFB was performed. Different amounts of sprouted oat flour (5–20% w/v) were homogenized with tap water and the soluble protein, β-glucan and phenolic contents of the resulting sprouted oat beverages were evaluated. A gradual increase in the content of these compounds was observed as the percentage of sprouted oat flour raised up to 18% (w/v). A percentage higher than 18% resulted in a semisolid matrix and no significant increase of soluble protein, β-glucan and phenolics was observed. Therefore, 18% (w/v) of sprouted oat flour was chosen as the optimum substrate amount to elaborate the beverage.

After optimization experiments, sprouted oat flour was mixed with tap water (18% *w*/*v*), sucralose (0.2% *w*/*v*) and salt (0.1% *w*/*v*) in a glass jar with a screw cap. The resulting suspension was shaken for 2 h at 22 °C in an orbital shaking incubator (140 rpm), then pasteurized (90 ± 2 °C for 25 min) and cooled down to room temperature. Afterwards, beverage was filtered through a sterile nylon cloth (200 μm diameter) intended for vegetable drinks elaboration (Alcavida, Barcelona, Spain). Then, sodium bicarbonate (0.35% *w*/*v*) and starter culture (0.7% *v*/*v*) were added to the filtered oat beverage and fermentation was carried out at 30 °C for 4 h and 140 rpm in an orbital-shaking incubator. Since sprouted oat flour exhibited a slight bitter taste most likely attributed to the activation of lipase activity during oat germination and release of fatty acids [23] and also to its high phenolic content [21], sodium bicarbonate (0.35% *w*/*v*) was added to counteract this undesirable flavor in SOFB. After fermentation, SOFB was distributed in sterile twist-off cap glass bottles (200 mL/bottle) and stored at 4 °C. A volume of 100 mL of SOFB was freeze-dried (Virtis Company, Inc., Gardiner, NY, USA) for further chemical analysis.

### 2.5. pH and Titratable Acidity

The value of pH was determined using a digital pH meter Basic 20 (Crison, Barcelona, Spain), previously calibrated with buffers at pH 4 and 7, according to manufacturer recommendations. Titratable acidity was determined by using a Titrator TL 7000 equipped with a pH-electrode (SI Analytics, Mainz, Germany) on 11 mL of SOFB mixed with 40 mL of distilled water. Titration was carried out with 0.1 N NaOH to a final pH of 8.2. Titratable acidity was expressed as g of lactic acid/100 mL of SOFB.

### 2.6. Organic Acids

Lactic and acetic acid contents were quantified by High Performance Anion Exchange Chromatography using an ion chromatographic system coupled to 800 Dosino dispenser, Bioscan module and a conductivity detector (Metrohm, Heriau, Switzerland). A 5 μm Metrosep organic acids column (4.0 × 250 mm, 5 μm) operated at room temperature using 0.5 mM sulfuric acid NaOH containing 15% of acetone as mobile phase at a flow rate of 0.5 mL/min was used to separate organic acids. The injection volume was 20 µL. Quantification of identified peaks was performed using HPLC grade standards of lactic and acetic acids. Data acquisition and processing were achieved by Metrodata IC Net 2.3 software. Results were expressed as mg/L of SOFB.

### 2.7. Water Holding Capacity

Water holding capacity (WHC) of SOFB was determined by centrifugation, according to the method described by Nionelli et al. [14]. The results were calculated according to the following equation:

% WHC = [(Sample weight *−* Expelled water)/Sample weight] × 100.

### 2.8. Microbiological Analysis

Enumeration of *L. plantarum* WCFS1 viable cells was performed by the pour plate technique in MRS agar (pH 6.2 ± 0.2, Condalab, Madrid, Spain). The results were expressed as log of colony forming units (Log CFU) per mL of SOFB. Microbiological analysis of SOFB was conducted by the pour plate technique according to the International Organization of Standardization (ISO) and French Standardization Association (AFNOR). The following microorganisms were enumerated: total aerobic mesophilic bacteria at 30 °C [24], *Enterobacteriaceae* [25], total coliforms, *Escherichia coli* β-glucuronidase positive [26], molds and yeasts [27], *Bacillus cereus* [28], *Salmonella* spp. [29] and *Listeria monocytogenes* [30]. The results were expressed as log of colony forming units (Log CFU) per mL of SOFB, with the exception of detection of *Salmonella* spp. and *Listeria monocytogenes*, where results were expressed as presence/absence in 25 mL of SOFB.

### 2.9. Total Protein, Thiamine, Riboflavin and β-glucan Content

Nitrogen content was determined in freeze-dried oat beverage by the Dumas method, using a nitrogen analyzer (LECO Corp., St. Joseph, MI, USA) [20]. A factor of 5.83 was used to convert nitrogen values to protein content, which was expressed as g/100 mL of SOFB. Vitamins B_1_ and B_2_ were extracted from freeze-dried oat beverage by a sequential acid and enzymatic hydrolysis and quantified by HPLC according to the method previously described [20]. The results were expressed as μg/100 mL of SOFB. The content of β-glucan was determined by the 1.3:1.4 mixed-linkage β-glucan kit (Megazyme, Wicklow, Ireland), according to the manufacturer instructions. The absorbance at 510 nm was measured in a microplate reader (Synergy HT, BioTek Instruments, Winooski, VT, USA). The results were expressed as mg/100 mL of SOFB.

### 2.10. Fatty Acids

Fatty acid profile was evaluated in freeze-dried SOFB by determination of fatty acid methyl esters (FAMEs) by gas chromatography on a gas chromatograph (7820A model, Agilent) equipped with a flame ionization detector (FID). A capillary fused silica column with a cyanopropyl-methylpolysiloxane stationary phase (HP-23cis trans FAME columns, 60 m × 0.25 mm i.d., 0.25 µm film thickness—Hewlett Packard, Palo Alto, CA, USA) was used for separation of fatty acids. The temperature of the column was set at 100 °C for 2 min, it was raised to 145 °C (8 °C/min), then it was maintained for 20 min, increased to 195 °C (5 °C/min) and held for 5 min, then increased to 215 °C for 5 min (5 °C/min) and, finally, it was raised to 230 °C (5 °C/min). The helium flow rate was 1 mL/min and the injection volume was 1 μL in the split mode 1:40. The temperature was set at 250 °C and 260 °C for detector and injector oven, respectively. The calibration of FAMEs was performed by relative response to tridecanoic acid (internal standard). Identification of FAMEs was performed by comparing their retention times with those of a FAMEs standard mixture (FAME 37 + PUFA N° 2 Animal Source + PUFA N° 3 Menhaden oil). Data acquisition and analysis were carried out by EZChrom Elite software. The results were expressed as g/100 g of total fatty acids.

### 2.11. Total Phenolic Compounds

Phenolic compounds were extracted from freeze-dried SOFB with methanol:HCl:water (80:0.1:19.9, *v*/*v*) and quantified by the Folin–Ciocalteu’s reagent according to Tomé-Sánchez et al. [19]. Gallic acid was used as standard and the results were expressed as mg of gallic acid equivalents (GAE)/100 mL of SOFB.

### 2.12. Oxygen Radical Absorbance Capacity (ORAC)

The antioxidant activity was determined by ORAC in methanol extracts as previously reported [20]. The fluorescence was determined on a Synergy HT microplate reader (BioTek, Winooski, VT, USA) at emission and excitation wavelengths of 520 nm and 485 nm, respectively. Trolox was used as standard and the results were expressed as mg of Trolox equivalents (TE)/100 mL of SOFB.

### 2.13. Estimation of SOFB Stability during Refrigerated Storage

The stability of SOFB during storage was evaluated at 4 °C for 20 days. Samples from SOFB were taken periodically (at 0, 5, 10, 15 and 20 days) during the storage period and the microbial quality, viability of the starter culture, pH, titratable activity, WHC and β-glucan content were analyzed.

### 2.14. Sensory Evaluation

A total of 16 non-trained panelists aged between 20 and 60 years recruited at the Institute of Food Science, Technology and Nutrition (ICTAN-CSIC) evaluated the acceptability of the SOFB developed. Sensory analysis was performed after SOFB storage at 4 °C for 2 days. A volume of 30 mL of beverage was served at a temperature of 4 °C in odor-free transparent cups to each panelist. The overall acceptability was rated based on color, aroma, taste, consistency and overall acceptability. A 9-point hedonic scale was used to evaluate each attribute, where 1 means “dislike extremely” and 9 means “like extremely”.

### 2.15. Statistical Analysis

The data are average of three replicates from three separate fermentations. Significant differences between data were evaluated by one-way analysis of variance (ANOVA) and post hoc Duncan’s multiple range test (*p* ≤ 0.05) using Statgraphics Plus software version 5.1 (Statistical Graphics Corporation, Rockville, MD, USA).

## 3. Results and Discussion

### 3.1. Physicochemical Characteristics of SOFB

The values of pH, titratable acidity, content of lactic and acetic acids and WHC of SOFB are shown in Table 1. SOFB exhibited a pH value (6.96) close to neutral pH while titratable acidity was 0.42 g of lactic acid/100 mL. A similar pH value was observed in an oat beverage after 4 h of fermentation with *L. plantarum* NCIMB 8826 [16]. However, lower pH and higher acidity values have been reported in other grain-based beverages obtained from oat, wheat and quinoa by lactic acid fermentation for 12–24 h [14,31]. The shorter fermentation time (4 h) and starter culture load and type used in the present study and the different grain substrate could partially explain the lower acidity of SOFB compared with those obtained in other studies. Moreover, the addition of sodium bicarbonate (0.35% *w*/*v*) before fermentation of sprouted oat beverage undoubtedly contributed to the high pH value observed in SOFB. In fact, previous studies performed by our group showed that the pH values (4.5–5) in the fermented beverage after 4 h of fermentation without sodium bicarbonate addition (results not shown) were close to values observed in the literature.

The contents of lactic and acetic acids were also quantified in SOFB (Table 1), since *L. plantarum* is a facultative heterofermentative bacteria that produces both organic acids by metabolizing carbohydrates [16]. The levels obtained (1634.4 and 89.5 mg/L for lactic and acetic acids, respectively) were in agreement with values reported in a barley malt beverage obtained by fermentation with *L. acidophilus* or *L. plantarum* for 4–6 h [32].

WHC of SOFB, which represents the ability of the beverage to hold all or part of its own water, was close to 35% (Table 1). This value was lower as compared to those reported in a beverage obtained from oat flakes fermented with a *L. plantarum* O9 [14]. The lower WHC observed in the present study can be attributed to the higher activity of cell-wall disintegrating enzymes (β-glucanase and xylanases) and free β-glucan content [33] in sprouted oat flour than in oat flakes used in the earlier study.

### 3.2. Viability of L. plantarum in SOFB

The inoculation of *L. plantarum* WCFS1 (0.7% *v*/*v*) to SOFB provided viable cell counts of 6.8 Log CFU/mL at the beginning of fermentation (results not shown). *L. plantarum* reached populations of approximately 9 Log CFU/mL after 4 h of fermentation at 30 °C (Table 2). These results confirm that oat substrate has nutrient content high enough to support the growth and metabolism of lactic acid bacteria, as earlier reported by other researchers [14,16,17]. Similar starter bacteria population was observed in a quinoa beverage obtained by fermentation with *L. plantarum* 0823, *L. casei* Q11 and *L. lactis* ARH74 (1% *v*/*v* of each strain) for 6 h at 30 °C [34], while lower viable counts were reported in oat after fermentation with *L. plantarum* NCIMB 8826, *L. acidophilus* NCIMB 8821 or *L. reuteri* NCIMB 11951 (1% *v*/*v*) for 4–8 h at 30 °C [16]. Our results evidence that *L. plantarum* WCSF1 showed high adaptability to sprouted oat flour. Previous research carried out by our group has revealed that sprouting enhanced the nutritional value of oat by increasing the content of proteins, several essential amino acids, total polyunsaturated fatty acids, riboflavin and minerals [20,21]. Moreover, the activation of α-amylases during oat sprouting causes the starch hydrolysis, yielding glucose [20], making sprouted oat flour an excellent substrate for *L. plantarum* WCSF1 growth. Furthermore, *L. plantarum* WCSF1 seems to be a suitable strain for oat fermentation, in the view of its faster growth at shorter fermentation times as compared with other LAB strains used for grain fermentation reported in the literature. The suitability of *L. plantarum* as starter culture for vegetable fermentations has been largely demonstrated [35,36] and can be attributed to its versatile metabolism, high adaptability to different environmental niches [22,37], and capacity of growth on plant materials with high content of phenolic compounds [38]. These properties can be attributed to its complex genome, one of the largest found among LAB. In fact, the genome of *L. plantarum* WCFS1 exhibits a large set of genes involved in sugar uptake and utilization that belong to the group of highly expressed genes, providing to this strain the ability to efficiently use a wide variety of carbon sources [37]. The presence of coding sequences related to carbohydrate use in the genome of WCFS1 strain not found in other *L. plantarum* strains [39] suggests that this strain may exhibit different metabolic pathways to complex carbohydrate fermentation compared to other strains. These findings might explain the good adaptation of this strain to sprouted oat substrate used in the present study.

### 3.3. Microbiological Quality of SOFB

Enumeration of microbial groups related with spoilage status and safety was performed in SOFB (Table 2). Aerobic mesophilic bacteria are used as indicator of spoilage status of foods and good practices throughout processing. However, the high counts observed for this bacterial group in SOFB can be attributed to *L. plantarum* WCFS1 starter culture, taking into account that the beverage was heat-treated to reduce microbial load and also that this strain is able to grow in PCA at 30 °C. In fact, the most abundant colonies grown in PCA were subjected to morphological and biochemical assays and it was confirmed that they corresponded to *L. plantarum* (results not shown). *Enterobacteriaceae* and total coliforms counts were also below the limit of detection, indicating the absence of fecal bacteria contamination from environmental sources. Similarly, the presence of molds and yeasts was not detected, confirming the adequate handling practices during beverage manufacture. The absence of detectable levels of pathogenic bacteria (*E. coli*, *B. cereus*, *Salmonella* spp. and *L. monocytogenes*) suggested the safety of SOFB.

### 3.4. Nutritional Characteristics of SOFB

SOFB showed a higher protein content (1.7 g/100 mL) (Table 3) in comparison with other fermented and non-fermented beverages obtained from vegetables, fruits and cereal-milk mixes and fruits [40,41]. β-glucan, compound that exerts a wide range of biological activities (antioxidant, anti-osteoporotic, cholesterol-lowering, antitumorigenic, immunomodulatory, anti-obesity and prebiotic) [42,43] was also present in considerable amounts (79 mg/100 mL) (Table 3). It should be noted that β-glucan content in SOFB is lower than that expected taking into account that sprouted oat contained 2 g β-glucan/100 g [21], results that can be attributed to the fact that a fraction of β-glucan is insoluble in water due to its entanglement in phenolic cross-linked pentosans (unextractable β-glucan) [44]. β-glucan levels in SOFB was lower than those reported by Angelov et al. [11] in oat-based fermented beverages containing 7% of non-germinated oat flour, results expected since during sprouting oat β-glucanases hydrolyze β-glucan, reducing its content.

Thiamine and riboflavin contents were also quantified in SOFB (Table 3), and the values were higher than those previously reported in other cereal fermented beverages such as kidney bean-based beverage [45] and oat and maize kefir-like beverages [15]. Nevertheless, riboflavin levels in SOFB were lower than those expected taking into consideration the levels observed in sprouted oat flour [21]. A study performed in oat-based foods has demonstrated that some *L. plantarum* strains that harbor the rib operon in their genome are able to produce riboflavin [46]. However, genome of *L. plantarum* WCFS1 strain contains an incomplete rib operon, necessary for riboflavin biosynthesis [47], and therefore this strain is unable to grow in absence of riboflavin. The reduction of vitamin B_2_ content in SOFB suggests that it was used *L. plantarum* WCFS1 growth and supports this hypothesis.

Table 4 collects the fatty acid composition of SOFB. Linolenic acid was the dominant fatty acid, following by oleic acid and palmitic acid unlike legume seeds such peanuts were oleic acid was the most abundant fatty acid [48]. The observed profile is consistent with that previously reported in sprouted oat flour [21]. The results indicate that polyunsaturated fatty acids dominate in SOFB, while saturated fatty acids were found in the lowest amount. There is convincing evidence that replacing SFA with PUFA decreases LDL cholesterol concentration and the total/HDL cholesterol ratio, thus reducing the risk of cardiovascular diseases [49]. Moreover, PUFA/SFA ratio recommended for a balanced nutrition is above 0.4 [50]. Taking into account these recommendations, SOFB has a favorable fatty acid composition (PUFA/SFA ratio: 2.1).

### 3.5. Content of Total Phenolic Compounds in SOFB

**Figure 1:**
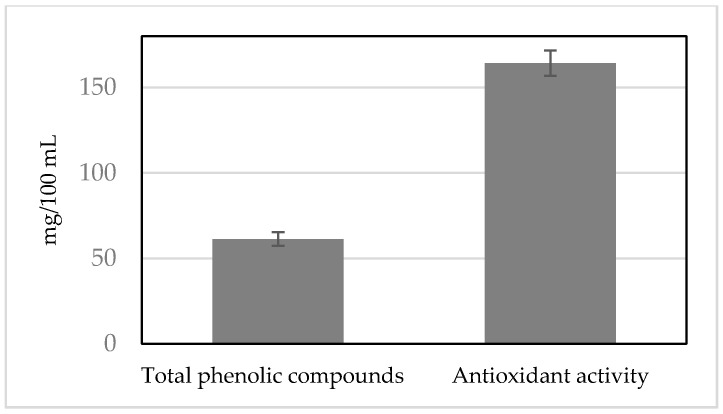
Content of total phenolic compounds (mg GAE/100 mL) and antioxidant activity (mg TE/100 mL) in SOFB.

SOFB exhibited a high TPC content (61.4 mg GAE/100 mL) (Figure 1) as compared to other vegetable fermented beverages produced from wheat bran and corn flour by spontaneous fermentation (190–257 mg ferulic acid/L) [51], and germinated/ungerminated barley, finger millet and mung bean fermented by *L. acidophilus* (2.04–2.48 mM GAE) [12].

Accumulative scientific evidence demonstrated that phenolic compounds counteract oxidative stress by sequestering oxidant nitrogen and oxygen species, transfer electrons to free radicals and activating antioxidant enzymes, thus contributing to the prevention of diabetes, cancer, cardiovascular diseases and obesity [52]. However, to exert their biological activity, phenolic compounds must be available in the target tissue. Recent studies have demonstrated that germination efficiently increased soluble phenolic content in cereals [18,19,20]. The bio-conversion of phenolic conjugated forms to the corresponding aglycones and release of bound forms to the corresponding free forms during lactic acid fermentation has been also well established [17,53]. Taking together, all these findings suggest that fermentation of sprouted oat is a promising approach to manufacture novel antioxidant cereals-derived foods enriched in bioaccesible phenolic compounds.

### 3.6. Oxygen Radical Antioxidant Activity (ORAC)

As shown in Figure 1, SOFB showed good antioxidant potential (164.3 mg TE/100 mL), which was more than 20-fold higher than that reported in an okara beverage fermented by *Lactobacillus rhamnosus* and *Bifidobacterium animalis* ssp. lactis Bb12 [54]. The high antioxidant activity in SOFB might be related with the release of soluble conjugated or insoluble bound phenolic compounds from oat cell wall by *L. plantarum* during fermentation, as previously observed in mulberry juice [55]. The high amount of phenolic compounds and PUFA, which are important natural antioxidants, in sprouted oat flour undoubtedly also contributed to the high ORAC values observed in SOFB. Since oxidative stress play a crucial role in the pathogenesis of a number of chronic diseases, daily consumption of SOFB could have a beneficial impact on health.

### 3.7. Acceptability of SOFB

**Figure 2:**
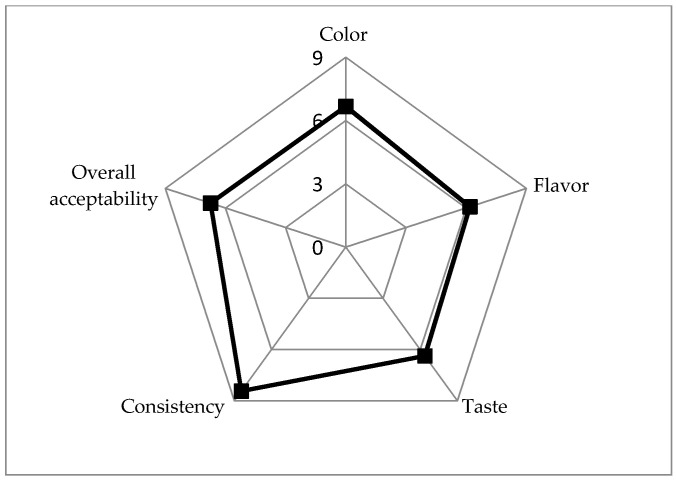
Hedonic test results for sensory analysis of SOFB.

Consumer perception is a crucial factor for the development of novel food products since it influences consumer willingness to purchase them. To evaluate the acceptability of SOFB by potential consumers, a hedonic scale sensory analysis was performed. The results are depicted in Figure 2. The novel fermented beverage produced was well accepted by the panelists, receiving all attributes scores higher than 6. Consistency was the sensory descriptor better scored, indicating that SOFB exhibited good homogeneity and texture. The sensory testing results obtained suggested that the mild-yellow color, acidic taste and oat-related flavor that characterized SOFB did not adversely affect its sensory perception, even though it constitutes a novel product. The hedonic ratings obtained for SOFB are higher than those previously reported for non-fermented obtained from chickpea, coconut, quinoa, soy, rice and oat [56,57,58] and plant-based yogurts [59]. The overall acceptability score obtained for SOFB (6.8) was very satisfactory considering that it constitutes an unfamiliar novel food and also that no sugar and flavorings were added. These results are consistent with previous studies showing that 73% of panelists would consume more plant-based foods if they had proven health benefits [56] and 84% of panelists are not afraid to eat products never eaten before [59].

### 3.8. Effect of Refrigerated Storage on SOFB Quality

#### 3.8.1. Viability of *L. plantarum* Starter Culture during Refrigerated Storage

**Figure 3:**
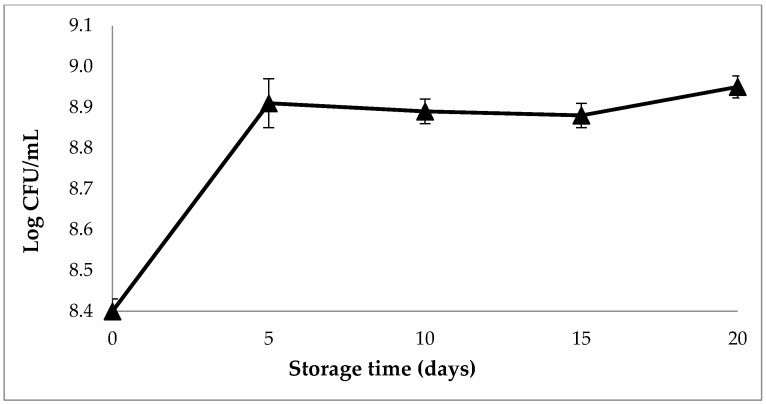
Viability of *L. plantarum* WCFS1 in SOFB during storage at 4 °C for 20 days.

Viability of *L. plantarum* WCFS1 in SOFB was evaluated for 20 days of storage at 4 °C (Figure 3). The viable counts of *L. plantarum* WFCS1 in SOFB increased 0.5 Log CFU/mL over the first 5 days of refrigerated storage and no further significant (*p* ≤ 0.05) enhancements were observed from 5 to 15 storage days. *L. plantarum* WFCS1 exhibited good stability over 20 days of refrigerated storage. These results demonstrate that the viability of the starter culture was not compromised and it was able to maintain its metabolic activity during the storage period. These findings are in agreement with results reported by Amanda and Choo [60] and Mesquita et al. [61], who observed a notable growth of *L. plantarum* and *L. paracasei* LBC 81 in watermelon juice and chickpea-coconut fermented beverages, respectively, during refrigerated storage for 10–14 days. Contrarily, several studies have reported the decrease of various lactobacilli species used as starters in different vegetable fermented beverages throughout the storage period [54,62]. According to the definition of the World Health Organization, probiotics are living microorganisms that when they are administered in adequate amounts confer a health benefit on the host [63]. Recommended properties for probiotic microorganism include survival in the gut, persistence in human gastro-intestinal tract, adherence to human epithelial cells, and proven safety for human consumption [37]. When administered in appropriate amounts, probiotic bacteria provide health benefits such as maintenance of a good microbiota composition, resistance against gastrointestinal infection by pathogens and also exhibit immunomodulatory effects [64]. The probiotic properties of *L. plantarum* WCFS1 in vitro and in vivo have been well documented [64]. Even though there is no general consensus on the recommended probiotic levels to confer beneficial health effects in the host, it has been suggested that the probiotic viability must be maintained at 10^6^–10^8^ CFU/mL until the food expiry date [65]. The counts of viable *L. plantarum* WCFS1 cells at the end of the storage period agree with this criterion, suggesting that SOFB can be considered a probiotic beverage.

#### 3.8.2. Changes in Content of Organic Acids, pH, Titratable Activity and WHC in SOFB during Refrigerated Storage

**Figure 4:**
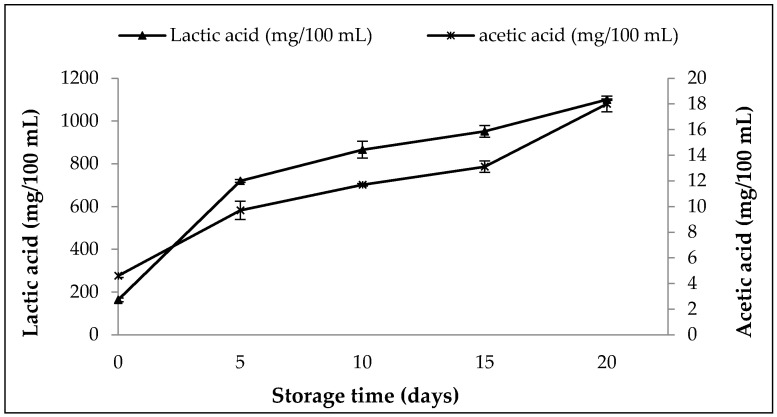
Changes in the content of lactic and acetic acids in SOFB during storage at 4 °C for 20 days.

Figure 4 illustrates the variations in the content of organic acids in SOFB during refrigerated storage. Both lactic and acetic acids increased steadily throughout the storage period, reaching values of 1100 mg/100 mL and 18 mg/100 mL for lactic and acetic acid, respectively, at the end of the storage period. It can be clearly observed that the production of both organic acids was more pronounced during the first 5 storage days, which is in accordance with the strong increase in *L. plantarum* WCFS1 viable cells observed during this period (Figure 3). The post-acidification of SOFB is the result of the fermentation of carbohydrates by the starter culture and indicates the active metabolic activity of *L. plantarum* WCFS1 at low temperatures. This phenomenon has been previously observed in soy-based fermented beverages produced with probiotic LAB [54].

**Figure 5:**
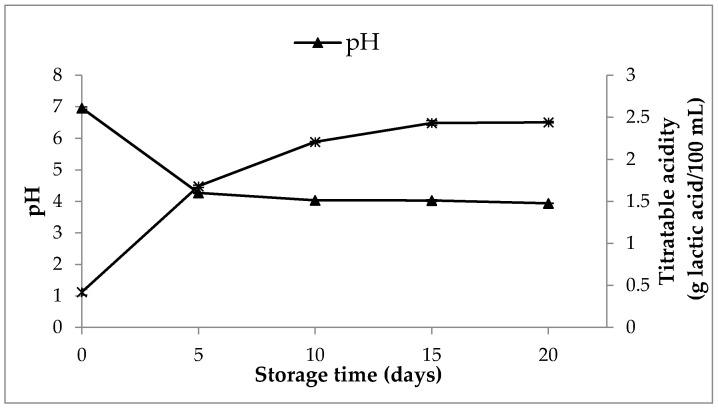
Variations in pH and titratable acidity of SOFB during storage at 4 °C for 20 days.

The pH value declined strongly during the first 5 days of storage, and no further variations in pH were observed (Figure 5). It should be noted that pH values were close to 4.0 during the storage period, in agreement with those previously observed in a symbiotic soy-based beverage stored for 28 days [54]. Accordingly to pH values, a sharp increase of titratable acidity occurred at the beginning of the storage period, and only slight increases of this parameter was found beyond 5 storage-days (Figure 5). A rapid acidification of SOFB at the beginning of storage is desirable due to its inhibitory effect against the growth of pathogenic and spoilage microorganisms [66]. The values of pH and titratable acidity observed are indirect indicators of starter metabolic activity during storage, and confirmed the results regarding viability of *L. plantarum* WCFS1 (Figure 3) and organic acid contents (Figure 4) observed in SOFB during the storage period.

Refrigerated storage did not significantly (*p* > 0.05) affect WHC of SOFB (Table 5), results that indicate that despite the increased acidification over the storage period there was no tendency to release water by SOFB, conversely to results observed by other authors in fermented dairy beverages [67].

#### 3.8.3. Changes in β-glucan Content of SOFB during Refrigerated Storage

β-glucan content in SOFB over 20 days of refrigerated storage is shown in Table 5. It is clearly noticeable that the levels of this compound remained constant during storage, results evidencing that *L. plantarum* WCSF1 did not ferment β-glucan in SOFB. Our results are in line with those previously observed in oat-based products fermented by different *Lactobacillus* strains [11,68]. Contrarily, Russo et al. [46] reported reductions in β-glucan content during fermentation and storage of oat-based foods due to the capability of the LAB strains to produce β-glucanases. The maintenance of β-glucan content during storage is of great importance to enhance the health-promoting properties of SOFB. Our results revealed that *L. plantarum* WCFS1 is a good candidate to be used as starter culture to achieve these purposes.

#### 3.8.4. Microbial Quality of SOFB during Refrigerated Storage

Results corresponding to the microbial analysis of stored SOFB (Table 5) revealed that neither spoilage microorganisms such as aerobic mesophilic bacteria, *Enterobacteriaceae*, total coliforms, molds and yeasts nor pathogenic bacteria such as *E. coli*, *B. cereus*, *Salmonella* spp. and *L. monocytogenes* were detected over the whole storage period. The growth of *L. plantarum* WCFS1 and subsequently acidification of SOFB during storage, as previously shown, are the main contributors to their stability and safety. Refrigerated storage for 20 days, therefore, maintained a good microbial quality of SOFB and ensured its safe consumption.

## 4. Conclusions

A novel gluten-free fermented beverage based on sprouted oat flour using the probiotic *L. plantarum* WCFS1 strain as starter culture was developed in this study. The results showed that sprouted oat flour is a suitable substrate that supports the fast growth and high viability of *L. plantarum* WCFS1 strain. The obtained beverage exhibited good physicochemical properties and microbiological quality and can be considered a good source of protein, β-glucan, thiamine, riboflavin and polyunsaturated fatty acids. The beverage presented good stability during refrigerated storage for 20 days, showing at the end of the storage period high viable populations of the probiotic starter culture, an adequate amount of β-glucan and good microbial quality that suggest that it can be safely consumed. SOFB is in line with current consumer demands regarding natural and wholesome gluten-free plant-based foods. Further studies will be conducted to evaluate the impact of this beverage on consumer’s health.

## Figures and Tables

**Table 1:** Physico-chemical characteristics of Sprouted Oat Fermented Beverage (SOFB).

	SOFB
pH	6.96 ± 0.04
Titratable acidity (g lactic acid/100 mL)	0.42 ± 0.01
Lactic acid (mg/L)	1634.4 ± 9.1
Acetic acid (mg/L)	89.54 ± 0.23
WHC (%)	34.93 ± 0.27

WHC: water holding capacity.

**Table 2:** Viable microbial counts (Log CFU/mL) in SOFB.

	SOFB
*L. plantarum* WCFS1	8.85 ± 0.004
Aerobic mesophilic bacteria	8.82 ± 0.005
*Enterobacteriaceae*	<1
Total coliforms	<1
Molds and yeasts	<1
*Escherichia coli*	<1
*Bacillus cereus*	<1
*Salmonella* spp.	ND *
*Listeria monocytogenes*	ND *

* ND: not detected (absence in 25 mL).

**Table 3:** Contents of protein, β-glucan, thiamine and riboflavin in SOFB.

	SOFB
Protein (g/100 mL)	1.67 ± 0.009
β-glucan (mg/100 mL)	79.0 ± 6.0
Thiamine (μg/100 mL)	676.0 ± 4.0
Riboflavin (μg/100 mL)	28.12 ± 1.04

**Table 4:** Fatty acid profile (g/100 g total fatty acids) of SOFB.

Fatty Acids	SOFB
14:0 (Myristic)	0.34 ± 0.009
16:0 (Palmitic)	19.18 ± 0.03
16:1n7 (Palmitoleic)	0.36 ± 0.005
18:0 (Stearic)	1.64 ± 0.006
18:1n7c (Asclepic)	1.01 ± 0.003
18:1n9c (Oleic)	32.10 ± 0.03
18:2n6c (Linoleic)	42.56 ± 0.03
18:3n3 (α-Linolenic)	1.58 ± 0.01
20:0 (Arachidic)	0.15 ± 0.01
20:1n9 (*cis*-11-Eicosenoic)	0.75 ± 0.003
20:5n3 (EPA)	0.16 ± 0.01
22:0 (Behenic)	0.16 ± 0.008
SFA	21.47 ± 0.04
MUFA	34.22 ± 0.02
PUFA	44.30 ± 0.03

MUFA: Monounsaturated fatty acids; PUFA: Polyunsaturated fatty acids; SFA: Saturated fatty acids.

**Table 5:** Water holding capacity (WHC, %), content of β-glucan (mg/100 mL) and viable counts (Log CFU/mL) of lactic acid bacteria, aerobic mesophilic bacteria, *Enterobacteriaceae*, total coliforms, molds and yeast, *E. coli*, *B. cereus*, *Salmonella* and *L. monocytogenes* in SOFB during storage at 4 °C for 20 days.

Storage Days					
	0	5	10	15	20
WHC	34.9 ± 0.3 ^a^	35.3 ± 0.1 ^a^	33.7 ± 1.0 ^a^	34.3 ± 0.6 ^a^	34.5 ± 0.5 ^a^
β-glucan	79.0 ± 6.0 ^a^	74.0 ± 1.0 ^a^	71.0 ± 2.0 ^a^	71.0 ± 1.0 ^a^	72.0 ± 3.0 ^a^
Aerobic mesophilic bacteria	8.82 ± 0.01 ^a^	8.91 ± 0.04 ^b^	9.04 ± 0.01 ^c^	8.93 ± 0.07 ^c^	9.02 ± 0.03 ^c^
*Enterobacteriaceae*	<1	<1	<1	<1	<1
Total coliforms	<1	<1	<1	<1	<1
Molds and yeasts	<1	<1	<1	<1	<1
*Escherichia coli*	<1	<1	<1	<1	<1
*Bacillus cereus*	<1	<1	<1	<1	<1
*Salmonella* spp.*	ND	ND	ND	ND	ND
*Listeria monocytogenes* *	ND	ND	ND	ND	ND

Data are the mean ± standard deviation of three replicates. Different superscript letters within a row indicate significant differences (one-way ANOVA, post hoc Duncan’s test *p* ≤ 0.05). * ND: not detected (absence in 25 mL).
